# Fluoxetine Treatment Abolishes the *In Vitro* Respiratory Response to Acidosis in Neonatal Mice

**DOI:** 10.1371/journal.pone.0013644

**Published:** 2010-10-26

**Authors:** Nicolas Voituron, Yuri Shvarev, Clément Menuet, Michelle Bevengut, Caroline Fasano, Erika Vigneault, Salah El Mestikawy, Gérard Hilaire

**Affiliations:** 1 Maturation, Plasticité, Physiologie et Pathologie de la Respiration, Unité Mixte de Recherche 6231, Centre National de la Recherche Scientifique - Université de la Méditerranée - Université Paul Cézanne, Marseille, France; 2 Department of Woman and Child Health, Karolinska Institute, Stockholm, Sweden; 3 Institute of Cytology and Genetics, Siberian Division of the Russian Academy of Sciences, Novosibirsk, Russia; 4 Department of Psychiatry, Douglas Hospital Research Center, McGill University, Québec, Canada; 5 Unité 952, Institut National de la Santé et de la Recherche Médicale, Paris, France; 6 Unité Mixte de Recherche 7224, Centre National de la Recherche Scientifique, Paris, France; 7 Université Pierre et Marie Curie, Paris, France; University of Giessen Lung Center, Germany

## Abstract

**Background:**

To secure pH homeostasis, the central respiratory network must permanently adapt its rhythmic motor drive to environment and behaviour. In neonates, it is commonly admitted that the retrotrapezoid/parafacial respiratory group of neurons of the ventral medulla plays the primary role in the respiratory response to acidosis, although the serotonergic system may also contribute to this response.

**Methodology/Principal Findings:**

Using *en bloc* medullary preparations from neonatal mice, we have shown for the first time that the respiratory response to acidosis is abolished after pre-treatment with the serotonin-transporter blocker fluoxetine (25–50 µM, 20 min), a commonly used antidepressant. Using mRNA *in situ* hybridization and immunohistology, we have also shown the expression of the serotonin transporter mRNA and serotonin-containing neurons in the vicinity of the RTN/pFRG of neonatal mice.

**Conclusions:**

These results reveal that the serotonergic system plays a pivotal role in pH homeostasis. Although obtained *in vitro* in neonatal mice, they suggest that drugs targeting the serotonergic system should be used with caution in infants, pregnant women and breastfeeding mothers.

## Introduction

From birth onwards, the neonatal mammal must be able to breathe and adapt its breathing activity to environmental changes and behaviors. Therefore a correct function of the ponto-medullary respiratory network is required at birth, not only for the elaboration of the respiratory rhythm but also for its adaptation to physiological needs. In neonates, the respiratory rhythm generator (RRG) is composed of two coupled, interacting networks: the preBötzinger complex (preBötC) which contains the primary rhythm generating neurons in brainstem slices [1], [2] and the parafacial respiratory group (pFRG). The pFRG neurons express the transcription factor Phox2b, display a pre-inspiratory discharge and play a major role in detecting CO2/pH changes and adjusting the RRG activity in neonatal and juvenile animals [3], [4]. In embryos, organized rhythmic activities emerge in the preBötC and pFRG as early as at embryonic day 15.5 and 14.5, respectively [5]–[7]. After birth, the embryonic pFRG forms the retrotrapezoid nucleus (RTN) with Phox2b glutamatergic neurons detecting changes in CO2/pH, receiving peripheral inputs from carotid bodies chemoreceptors, and controlling the respiratory network [8]–[10]. The RTN/pFRG neurons are severely depleted in transgenic mouse model of the Central Congenital Hypoventilation Syndrome, a rare disease defined by the lack of CO2/pH responsiveness and RRG automaticity during sleep [11].

Although the RTN/pFRG neurons have a crucial role in ventilatory responses to CO2/pH changes in neonates, the serotonin (5-HT) neurons also contribute to the maintenance of blood gas homeostasis [12]–[14]. As recently reviewed, the 5-HT neurons are involved in respiratory function and dysfunction [14], they synaptically contact the RRG [15], modulate the activity of the maturing RRG [16], are intrinsically chemosensitive *in vitro*, are stimulated by hypercapnia *in vivo*, and their disruption alters the ventilatory response to CO2/pH changes [12], [17], [18]. In a transgenic mouse model of Prader-Willi syndrome, a rare disease with complex symptoms including frequent apnoeas during sleep and blunted ventilatory responses to CO2/pH, the medullary 5-HT levels are abnormally increased at birth and the ventilatory responses to CO2/pH reduced [15]. In addition, an altered 5HT system and insufficient ventilatory responses to CO2/pH and/or hypoxia during sleep might contribute to sudden infant death syndrome (SIDS), the main cause of death in infants in industrialized countries [14], [18]–[21].

The present work aimed at a further elucidation of the role of endogenous 5-HT in the RRG response to CO2/pH in neonatal mice. Using *en bloc* medullary preparations where the isolated RRG still functions *in vitro* and responds to CO2/pH changes, we show that a pre-treatment with fluoxetine, a blocker of the serotonin transporter (SERT) and 5-HT re-uptake, abolishes the RRG responses to CO2/pH. Using mRNA hybridization and immunohistology, we also report SERT mRNA expression and a few 5-HT neurons in the vicinity of the RTN/pFRG area. As fluoxetine is a commonly used antidepressant known to increase endogenous levels of 5-HT [22], we suggest a cautious use of pharmacological treatments targeting the 5HT system in infants, pregnant women and breastfeeding mothers.

## Materials and Methods

### Ethics Statement

The experiments were performed on neonatal mice (n = 48) born from C57BL6 mice (Charles River Laboratories; l'Arbresle, France) housed with food and water ad libitum and were conducted in agreement with the European Communities Council Directive (86/609/EEC) (permit number 13–426 to Nicolas Voituron; approved by Direction Départementale des Services Vétérinaires - Préfecture des Bouches du Rhône, France).

### Surgery and electrophysiology

The medulla and cervical cord of neonatal mice (n = 42; postnatal days 1–3) were dissected, placed in a 2 ml *in vitro* recording chamber and superfused (>2 ml per min) with artificial cerebro-spinal fluid (aCSF), as reported previously [15]. Under these conditions, the isolated RRG continued to function, producing rhythmic bursts on phrenic roots for long periods of time. The 4^th^ cervical ventral root containing the axons of phrenic motoneurons was sucked in a glass micropipette. Its electrical activity was filtered (100–3000 Hz), amplified (×5000), integrated (time constant 100 ms), digitized and stored (1 Khz; Spike 2 data system; Cambridge Electronic Design, UK).

As reported previously [7], [23], two different aCSF, bubbled with carbogene and maintained at 27°C, were used: a normal aCSF (pH = 7.4) and an acidified aCSF (pH = 7.1), and pH values were regularly measured (pHmeter P107, Consort, Bioblock Scientific). The composition of the normal aCSF, also termed below aCSF(7.4), was (in mM): 129.0 NaCl, 3.35 KCl, 21.0 NaHCO3, 1.26 CaCl2, 1.15 MgCl2, 0.58 NaH2PO4, and 30.0 D-glucose. The composition of the acidified aCSF, also termed below aCSF(7.1), was similar but NaHCO3 was reduced to 10.0 mM [7], [23]. This protocol already used by us [7] and others [23] to impose reproducible acidosis to en bloc medullary preparations without bubbling gases containing higher levels of CO_2_ and lower levels of O_2_ in the superfusate, was used to examine the direct effects of acidosis on the activity produced by the isolated respiratory network. In a first set of experiments, the RRG response to acidosis was analyzed by subjecting preparations to 5 or 10 min application of aCSF(7.1). In a second set of experiments, the effect of pre-treating the preparations with aCSF(7.4) containing fluoxetine (10 µM or 50 µM; 20 min) was investigated on the RRG response to acidosis. Fluoxetine is a well-known antidepressant of common clinical use that blocks the serotonin transporter (SERT), the blockade of the 5-HT reuptake increasing the 5-HT endogenous levels and potentiating the 5-HT effects at the synaptic cleft [22]. After fluoxetine pre-treatment, the RRG response to acidosis was investigated by subjecting the preparations to aCSF(7.1) containing the same amount of fluoxetine for 5 min. In a third set of experiments, the RRG response to acidosis was investigated after pre-treatment with aCSF(7.4) containing 5-HT (1 µM or 5 µM; 20 min) to mimic the effect of fluoxetine by increasing the extracellular level of 5-HT. After 5-HT pre-treatment, the RRG response to acidosis was analyzed by subjecting the preparations to aCSF(7.1) containing the same amount of 5-HT for 5 min. All drugs were purchased from Sigma (Sigma-Aldrich, Saint-Quentin Fallavier, France) and dissolved in aCSF.

For each individual experiment (n = 42), the phrenic burst frequency (PBf) was measured every min. The 5 min period under aCSF(7.4) prior to any drug application was used to define the control PBf (100%) and the changes in PBf induced by application of the modified aCSF were expressed in % of control PBf. Standardized experiments were repeated on different preparations from different litters and only one trial was performed per preparation. The mean changes in PBf were averaged min after min from different preparations and the results were given as mean ± SEM. As in previous reports [15], [16], [24], [25], the figures present the mean PBf changes as % of the control PBf, whereas the statistical analysis of the changes in PBf was performed on absolute values (cycle per min, c.min^−1^) using a student's paired *t*-test to compare the PBf during acidosis to the mean PBf calculated during the five min prior to the acidosis tests. The level of statistical significance was set at p<0.05.

### SERT mRNA expression and 5-HT neurons analysis

To analyze SERT mRNA expression in neonatal mice (n = 3), brains were dissected at postnatal day 6 and frozen in isopentane at −30°C. Sections (10 µm) were prepared with a cryostat at −20°C, thaw-mounted on glass slides and stored at −80°C until usage. Regional mRNA hybridization *in situ* for the SERT sequences (*Mus musculus*, accession numbers: NM_010484.2) was performed as already described [26] with the following antisens oligonucleotide:

5′- GGAGGAGATGAGGTAGTAGAGCGCCCAGGCTATG-3′,

5′- GAACAGGAGAAACAGAGGGCTGATGGCCACCCAG-3′,

5′-CTCAGCAGGTGACGTGGAATGGAGTGTCCAGGTG-3′,

5′- AGAACCAAGACACGACGACGGCCTCGATGAGAGC -3′

and 5′- CCCACACCCCTGTCTCCAAGAGTTTCTGCCAGTTGG-3′, using Helios oligo design software (http://www.heliosbioscience.com, Helios Biosciences, France) [26]. Negative controls were performed using labeled sense oligonucleotides. In brief, oligonucleotides were labeled with [^35^S]-dATP, using terminal transferase (Amersham Biosciences, UK), to a specific activity of 5×10^8^ dpm/µg. Sections were covered with 100 µl of an hybridization of Helios medium [26] and 3–5×10^5^ dpm of each labeled oligonucleotide. Samples were incubated overnight at 42°C, washed and exposed to BAS-TR Fuji Imaging screens (Fuji Film Photo Co., Tokyo, Japan) for 4–5 days. Screens were then scanned with a Fuji Bioimaging Analyzer BAS-5000 (Fuji Film Photo Co., Tokyo, Japan).

To analyze 5-HT neurons expression, brainstems were dissected at postnatal day 3 (n = 3) and fixed in 4% paraformaldehyde dissolved in phosphate-buffered saline (PBS; 0.1 M phosphate buffer at pH 7.4+0.15 M NaCl). Free-floating serial coronal sections (70 µm thickness; vibroslicer Campden Instruments, Longhborough, UK) were first incubated for 30 min at room temperature in a blocking solution (BS) containing 1% normal goat serum and 0.3% w/v Triton X-100 in PBS, followed by overnight incubation at 4°C in an anti-5HT antibody (Sigma), diluted 1∶1,000 in BS. After rinsing in PBS, the sections were incubated for 4 hours at room temperature in a goat anti-mouse IgG Rhodamine-conjugated (Jackson ImmunoResearch, West Grove, PA), diluted 1∶200 in PBS containing 1% normal goat serum, rinsed again, and incubated overnight at 4°C in a mouse PAP complex (Sigma), diluted 1∶100 in PBS. On the following day, the sections were first rinsed and pre-treated for 20 min in a cobalt (0.5%) solution and rinsed; then the PAP complex was developed with 3, 3′-diaminobenzidine (DAB 0.04%) and hydrogen peroxide (0.01%). After rinsing in cold PBS, the sections were mounted on gelatin-chromalin coated slides, dried overnight, stained with neutral red (1% in H_2_O), dehydrated in a graded series of ethanol (70%, 80%, 95%, and 100%), cleared in xylene, coverslipped with DPX, investigated in transmitted light, and photographed.

## Results

**Figure 1:**
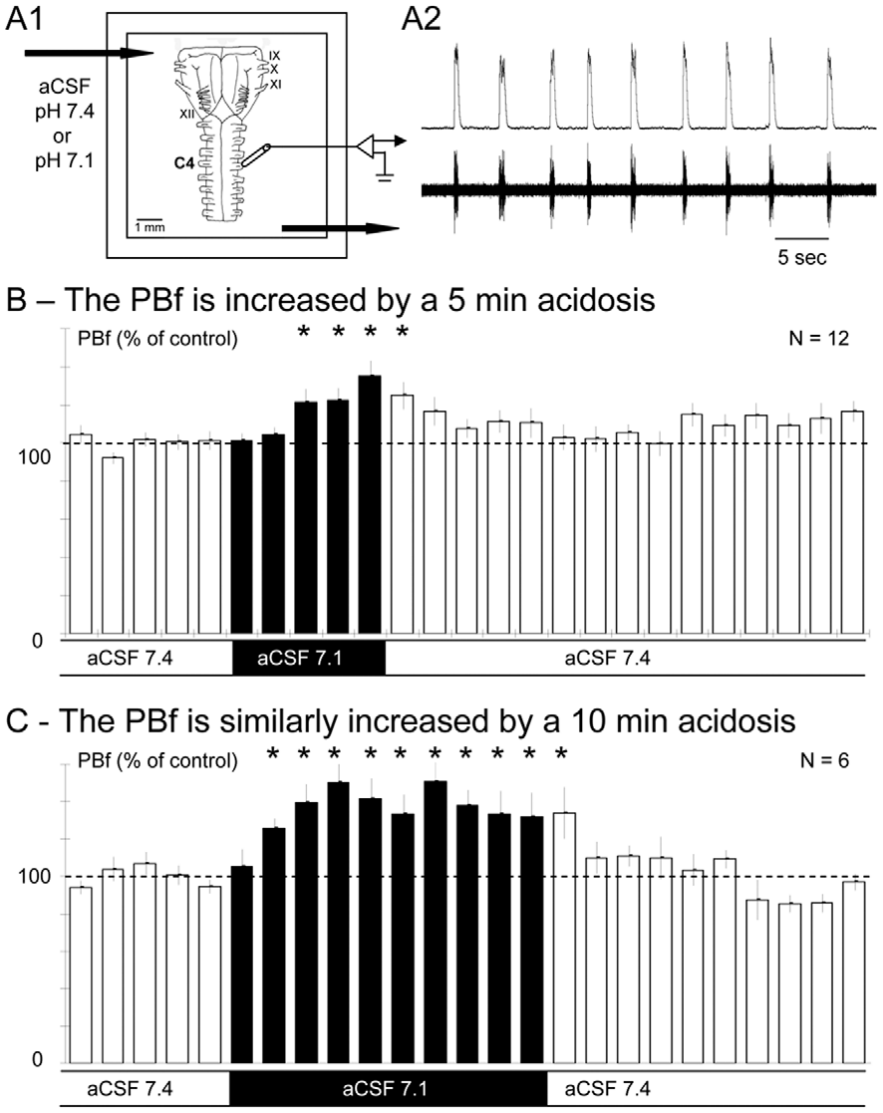
Acidosis increases the phrenic burst frequency of en bloc medullary preparations. A – Schematic presentation of the *en bloc* preparation of neonatal mice (A1) and example of raw and integrated phrenic bursts (bottom and top traces, respectively) produced by the isolated respiratory rhythm generator (RRG) on the C4 ventral roots of *en bloc* preparations (A2). B – Columns of the histogram show the mean (and SEM) phrenic burst frequency (PBf; expressed as % of the control PBf) measured every min (one column  = 1 min) in 12 *en bloc* preparations when the control aCSF(7.4) (white columns) superfusing the preparations was replaced by aCSF(7.1) for 5 min (black columns). Note that acidosis significantly increased the PBf (asterisks indicate a p<0.05 statistical difference). C- As in B but aCSF(7.1) application for 10 min to 6 other preparations. Note that the PBf reached a plateau from the 5^th^ to the 10^th^ min of aCSF(7.1) application.

The isolated RRG generated rhythmic phrenic bursts with stable amplitude and frequency (Fig. 1A) and the mean PBf under aCSF(7.4) containing no drugs (10.0±0.7 c.min^−1^; n = 42) was fully consistent with previously reported values [15].

### Acidosis increases the PBf of en bloc medullary preparations

To analyze the PBf changes induced by acidosis, we measured the control PBf every min in twelve preparations superfused with aCSF(7.4) for at least 20 min followed by superfusion with aCSF(7.1) for five min. As shown in Fig. 1B, the mean PBF did not change in the first two min of acidosis, after which it significantly increased to reach 133±8% of the control PBf during the last min of acidosis. When superfusion with aCSF(7.4) was resumed, the PBf recovered control values within two min. In a second set of experiments, six other preparations were also subjected to aCSF(7.1) but the application was maintained for ten min instead of five min to know whether longer period of acidosis induced more marked changes in the PBf (Fig. 1C). The PBf did not significantly change in the first min of acidosis, slightly but significantly increased at the 2^nd^ min, reached 142±10% at the 5^th^ min and thereafter remained at a plateau until the 10^th^ min of acidosis (132±13% of control) when superfusion with aCSF(7.4) was resumed, the PBf returning to control values.

Neither five nor ten min of acidosis significantly affected the mean amplitude of the integrated PB (data not shown). As five and ten min of acidosis similarly increased the PBf, data corresponding to the PBf changes induced by the first five min of acidosis in the two types of experiments (five and ten min of acidosis) were pooled together. On the whole sample of 18 preparations, acidosis always increased the PBf. It had no significant effects on the mean PBf during the first min, weakly but significantly increased it from the second min (112±4%) and raised the PBf up to 137±6% of the control at the 5^th^ min. No significant relationship was found between the PBf increment induced by acidosis at the 5^th^ min and the control PBf prior to acidosis (r = 0.312; df = 16; p>0.1). In addition, no significant difference was observed between the mean PBf increment induced by acidosis at the 5^th^ min in preparations displaying the highest (13.1±0.6 c.min^−1^, n = 5) vs. the lowest (5.9±0.2 c.min^−1^, n = 5) control PBf (mean PBf increment: 3.7±1.4 c.min^−1^ and 2.3±0.7 c.min^−1^, respectively; P>0.2). Therefore, acidosis increased the PBf in all the preparations, even when the control PBf was high. Because the five-min application of acidified aCSF was more likely to reflect the physiological breathing responses to transient blood acidosis than the ten-min application, we only tested below the PBf changes induced by five min of acidosis.

### Excess of 5HT abolishes the PBf response to acidosis

**Figure 2:**
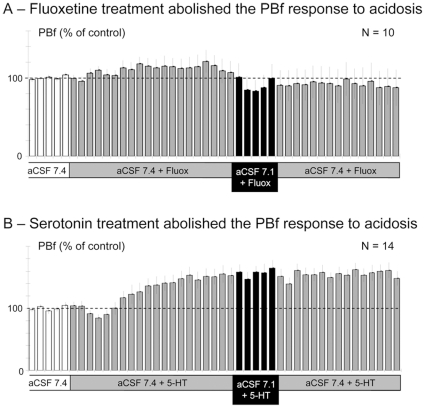
Fluoxetine and serotonin pre-treatments abolish the increase of phrenic burst frequency induced by acidosis. **A** - Columns of the histogram show the mean (and SEM) PBf (expressed as % of the control PBf) measured every min (one column  = 1 min) in 10 preparations when the control aCSF(7.4) (white columns) superfusing the preparations was first replaced by aCSF(7.4) containing fluoxetine (10–25 µM) for 20 min to block the serotonin transporter SERT (grey columns) and thereafter by aCSF(7.1) containing the same amount of fluoxetine for 5 min (black columns). Note that pre-treatment with fluoxetine did not significantly increase the PBf but abolished the PBf increase under acidosis. **B** - PBf changes (expressed as % of control) every min (one column) but for 14 preparations superfused with control aCSF(7.4) (white columns), aCSF(7.4) containing serotonin, 5-HT (1–5 µM) for 20 min (grey columns) and thereafter aCSF(7.1) containing the same amount of 5-HT for 5 min (black columns). Note that pre-treatment with 5-HT significantly increased the PBf by about 50% and, similarly to fluoxetine treatment, abolished the PBf increase under acidosis.

In a second set of experiments, preparations were subjected to pre-treatment with aCSF containing fluoxetine, a well-known SERT blocker known to increase 5-HT endogenous levels [22], prior to investigating the PBf responses to acidosis. As applications of fluoxetine at large concentration (100 µM) were reported to double the mean PBf [16], we used weaker concentrations to avoid marked increases of the resting PBf. Indeed, pre-treatments with fluoxetine at 10 µM (n = 6) and 50 µM (n = 4) did not significantly affect the resting PBf at either concentrations and therefore data were pooled (n = 10). As shown in Fig. 2A, subjecting the preparations to aCSF(7.4) containing fluoxetine for 20 min did not significantly increase the mean PBf during the last five min of fluoxetine (113±6% of the control; p>0.1). Then, to investigate the effect of fluoxetine pre-treatment on the PBf response to acidosis, the aCSF(7.4) containing fluoxetine was replaced by aCSF(7.1) containing the same amount of fluoxetine for 5 min. Acidosis slightly but non-significantly decreased the PBf, the PBf being similar to control at the 5^th^ min. Thus, pre-treating *en bloc* medullary preparations with the SERT blocker fluoxetine abolished the PBf response to acidosis.

To mimic the fluoxetine-induced increase of 5-HT levels, fourteen other preparations were subjected to aCSF(7.4) containing weak concentrations of exogenous 5-HT for 20 min. Two different concentrations of 5-HT were used, 1 µM (n = 7) and 5 µM (n = 7). After 20 min of application, they both slowly and significantly increased the PBf by around 50% and similarly altered the PBf response to acidosis; therefore data were pooled (n = 14). As shown in Figure 2B, aCSF(7.4) containing 5-HT slowly and significantly increased the PBf up to a plateau at 151±6% of the control during the five last min of 5-HT pre-treatment. Thereafter, applying aCSF(7.1) containing the same amount of 5-HT for 5 min to 14 preparations either reduced the PBf (83±7% of control; n = 6) or increased it (130±10% of control; n = 8). On the whole sample (n = 14), acidosis after 5-HT pre-treatment did not significantly affect the PBf at the 5^th^ min (112±9% of control; p>0.1). No significant relationship was found between the PBf responses to acidosis and the control PBf either prior to (r = −0.174; df = 12; p>0.1) or after 5-HT pre-treatment (r = −0.346; df = 12; p>0.1). In addition, no significant difference was observed between the PBf response to acidosis in preparations displaying the highest (22.7±2.4 c.min^−1^; n = 5) vs. the lowest (12.2±0.7 c.min^−1^; n = 6) PBf under 5-HT (mean PBf response: 0.2±0.9 c.min^−1^ and 1.4±1.6 c.min^−1^, respectively; P>0.2).Then, exposure of preparations to exogenous 5-HT abolished the PBf response to acidosis, even when the PBf was moderately increased by 5-HT exposure.

### SERT mRNA expression and 5-HT neurons in the RTN/pFRG area

**Figure 3:**
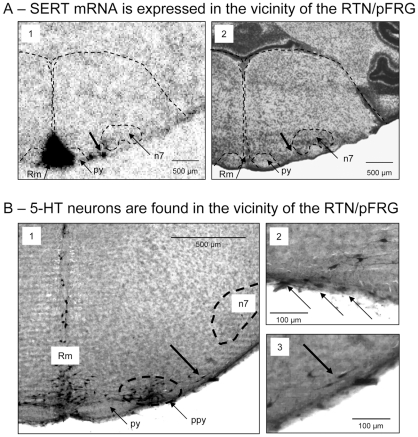
SERT mRNA and 5-HT-containing neurons in the vicinity of the RTN/pFRG of neonatal mice. A: Two serial coronal sections passing through the RTN/PFRG area of a neonatal mouse show SERT mRNA expression (A1) and Cresyl Violet staining (A2). Anatomical limits drawn from A2 Cresyl Violet section have been superimposed on A1 section (dotted line). Note SERT mRNA is expressed in three areas, the median Raphe Magnus (Rm), the para-pyramidal group (ppy) lateral to the pyramid tract (py) and a lateral spot (arrow) located in a medio-ventral position from the facial motor nucleus (n7), as defined from Cresyl Violet staining (in A2). B- 5-HT neurons in the RTN/pFRG area of a neonatal mouse. Note 5-HT neurons in the median raphe magnus (Rm), the parapyramidal group (ppy, doted circle) lateral to the pyramidal tract (py) and the lateral spot (arrow in B1) in the medio-ventral location of the facial nucleus (n7). In B2 and B3, note the superficial location of some 5-HT neurons (arrows). B3 is an enlargement of B1.

To decipher the mechanisms through which the SERT blocker fluoxetine abolished the PBf response to acidosis, we examined possible fluoxetine targets with *in situ* hybridization for SERT mRNA in 10 µm medullary sections of three neonatal mice. We identified the brainstem section level from observation of adjacent sections stained with Cresyl Violet. As previously [7], we defined the RTN/pFRG area as located below the caudal part of the facial motor nucleus (n7), rostral to the appearance of the nucleus ambiguous. SERT mRNA was constantly found expressed in three distinct ventral spots of medullary sections cut at the RTN/pFRG level (Fig. 3A). First, SERT mRNA expression formed a median, large and heavily stained triangle, possibly delimiting the raphe magnus (Rm in Fig. 3). Second, SERT mRNA expression was also found at a more lateral spot, about 500 µm from the midline, just lateral to the pyramidal tract (py), in an area possibly corresponding to the para-pyramidal subgroup of 5-HT neurons (ppy in Fig. 3). Third, a distinct and small spot of SERT mRNA was observed 200–300 µm laterals from the ppy, ventral to the medial part of the facial nucleus (n7). This small lateral spot was observed in the three studied neonatal mice (arrows in Fig. 3).

The SERT mRNA spot in the vicinity of the RTN/pFRG of neonatal mice was consistent with the dense 5-HT innervation to the RTN/pFRG described in rats [27]–[29]. However, as SERT mRNA expression could only originate from 5-HT somata and not 5-HT terminals, we used antibody against 5-HT to seek for 5-HT-containing neurons in sections passing at the RTN/pFRG level. We clearly identified three distinct groups of 5-HT neurons (Fig. 3B1) forming the medial raphe magnus, the lateral ppy column and a lateral group of a few scattered 5-HT neurons. The latter 5-HT-containing neurons were located very close to the ventral surface and medial to the n7 (Fig. 3B2, B3) and were highly likely at the origin of the most lateral SERT mRNA spot found in the vicinity of the RTN/pFRG area.

Asides from the RTN/pFRG area, our *in situ* hybridization studies revealed a very dense expression of SERT mRNA in all the median raphe nuclei and all along the ppy column of 5-HT neurons. At the caudal medullary level, SERT mRNA expression was also found in the respiratory-related areas of the ventro-lateral medulla, below the nucleus ambiguous, but not in the dorsal medulla (data not shown).

## Discussion

In neonatal mice, we report that the PBf response to acidosis is abolished by pre-treatment with the SERT blocker fluoxetine and that SERT mRNA and 5-HT neurons are found in the vicinity of the RTN/pFRG area. These new results are of main importance since they argue for a pivotal role of the 5-HT system in the PBf response to acidosis and suggest that altered 5-HT metabolism in infants may affect the breathing response to acidosis.

### Basic aspects of the PBf responses to acidosis

In medullary preparations of neonatal mice, acidosis increases the PBf by about 40% without affecting the phrenic burst amplitude, in agreement with previous reports in neonatal rats [30] and foetal mice [7]. Compelling evidence exist that the neonatal RTN/pFRG plays a main role in the PBf response to acidosis [3], [4], [8]–[10]. However, the one min latency of the PBf response appears to be long since the RTN/pFRG chemoreceptors are close to the ventral surface and rapidly detect CO2/pH changes [28]. The RRG responds in a few seconds when the RTN/pFRG neurons are stimulated by direct photo-activation *in vivo* [31] or direct CO2/pH stimulation *in vitro* [32]. The kinetics of the PBf responses to acidified aCSF applications have not been previously documented in details but latencies in a min range are commonly illustrated [30], [33], [34]. Aside the superficial RTN/pFRG chemoreceptors, several groups of chemoreceptors are described in discrete brainstem areas, such as the pontine catecholaminergic A5 and A6 areas, the ventrolateral medulla and the raphe areas [33], [35]. Indeed, the 5-HT neurons play a crucial role in respiratory function and dysfunction [14]: they are intrinsically chemosensitive [12], synaptically contact the respiratory neurons [15] and exert a facilitatory modulation on the RRG via a release of endogenous 5HT and activation of 5-HT1A receptors [16], [36]. Although some 5-HT neurons are very close to the ventral surface of the medulla, most are located deeper in the brainstem than the superficial RTN/pFRG chemoreceptors and may require a longer latency to detect acidosis. As five and ten min acidosis applications similarly affect the PBf, we speculate that the PBf response to acidosis we have analyzed reflects the integrated response of the whole medullary chemosensitive system rather than the solely response of superficial RTN/pFRG chemoreceptors.

### Basic aspects of the fluoxetine abolition of the PBf response to acidosis

Fluoxetine blocks SERT and increases the 5-HT endogenous level and the 5-HT availability at the synaptic cleft [22], [37]. Correlatively, fluoxetine potentiates the facilitation of the RRG by endogenous 5-HT, doubling the resting PBf when applied at 100 µM [16]. Herein applying fluoxetine at weaker concentrations did not significantly increase the PBf but totally abolished the PBf response to acidosis. Mimicking the fluoxetine-induced increase in 5-HT levels with exogenous 5-HT application significantly increased the PBf and also totally abolished the PBf response. The abolition of the PBf response by 5-HT applications was unlikely caused by the increased resting PBf since no significant relationship was found between the resting PBf and the magnitude of the PBf response to acidosis under normal aCSF as well as under aCSF containing 5-HT.

Our histological results in neonatal mice show SERT mRNA expression in all areas known to contain 5-HT neurons as well as in the vicinity of the RTN/pFRG area where we also report 5-HT-containing neurons. In the RTN/pFRG of neonatal rats, SERT immunoreactivity and 5-HT receptors expression has been reported, such a dense 5-HT innervation forming a possible anatomical substrate for 5HT-dependent control of RTN/pFRG chemoreceptors [27]–[29]. In neonatal mice, the neurons expressing the SERT mRNA and containing 5-HT in the vicinity of the RTN/pFRG area may be viewed as a lateral subcluster of 5-HT neurons from the ppy column but it cannot be excluded that they are not actual 5-HT neurons. During development, SERT expression can transiently occur in some glutamatergic non-5-HT neurons allowing them to take up and store 5-HT released from neighbouring terminals [38]. Further experiments are required to examine whether the SERT-expressing and 5-HT-containing neurons in the vicinity of the RTN/pFRG area are actual 5-HT neurons able to synthesize 5-HT or glutamatergic RTN/pFRG neurons expressing SERT and up-taking 5-HT. Nevertheless, fluoxetine blockade of 5-HT uptake may affect the 5-HT levels, the pH response of brainstem chemoreceptors and the RRG activity and responsiveness. Application of 5-HT to RTN/pFRG chemoreceptors increases their baseline level but not the magnitude of their response to pH [28]. Thus, the abolition of the PBf responses to acidosis by fluoxetine cannot be simply explained by a decreased chemosensitivity of RTN/pFRG neurons and it possibly reflects more complex, 5-HT dependent mechanisms affecting the whole chemosensitive system.

Within the maturing CNS, endogenous 5-HT acts through a plethora of receptors subtypes to modulate maturational processes, synaptic mechanisms and neuronal excitability [14], [38]. Similarly 5-HT exerts multiple effects on the neonatal respiratory network, facilitating the RRG via 5-HT1A receptors, depolarizing and firing the phrenic motoneurons via 5-HT2A receptors and reducing the transmission of the respiratory drive to motoneurons via 5-HT1B receptors [14], [16], [24], [25], [36], [39]. An excess of endogenous 5-HT affects the expression of 5-HT1A receptors, the RRG modulation by 5-HT [36], [40] and may also affect non-5HT systems known to modulate the RRG such as the catecholaminergic system [41], [42]. In addition, 5-HT may affect the gap junction coupling. Gap junction coupling occurs between cultured chemoreceptors [43] as well as between chemoreceptors of discrete brainstem areas such as the locus coeruleus [44], the raphe [45] and the RTN/pFRG [46]. Frequent gap junctions contribute to the synchronous firing of raphe neurons [45] and may lead the brainstem chemoreceptors to behave as a syncytium [47]. Blockade of gap junctions reduces the *in vivo* RTN/pFRG chemosensitivity [48] and the *in vitro* resting PBf [49]. Interestingly, the gap junction coupling is reduced by excess of 5-HT [50], [51]. We therefore speculate that the fluoxetine-induced excess of 5-HT reduces the gap junction coupling between brainstem chemoreceptors and that a disorganized chemoreceptor drive to the RRG during acidosis may affect the PBf response.

Thus, the fluoxetine-induced excess of 5-HT may directly (via the 5-HT system) and indirectly (via other neurotransmitter systems) affect the RRG responsiveness to acidosis as well as the elaboration of the chemoreceptor drive. Although further experiments are required to decipher these different, non-exclusive mechanisms, data from several lines of transgenic mice further support a link between 5-HT and RRG response to CO2/pH. Indeed, genetically-induced alterations of the 5-HT system in mice affect the RRG responses to CO2/pH, as reported in SERT knockout mice [37], [52] and lmx1b and Pet-1 knockout mice [53], [54]. In mouse model for Prader-Willi syndrome, a rare disease with blunted breathing responses to CO2/pH, neonates have excess of 5-HT in the medulla and reduced breathing response to CO2/pH [15]. In mouse model for the Central Congenital Hypoventilation Syndrome, the respiratory responses to pH are abolished, the number of 5-HT neurons is normal but nothing has been reported about 5-HT metabolism yet [11].

### Translational aspects of 5-HT and breathing responses to acidosis in infants

SIDS, the main cause of infant death in industrialized countries, might result from developmental abnormality of the 5-HT system leading to altered breathing responses to CO2/pH during sleep [18]–[21]. Alterations of 5-HT metabolism, number of 5-HT neurons, SERT expression and 5-HT receptors expression have been reported in SIDS and a subset of SIDS infants has genetic polymorphisms impacting the 5-HT system [14], [18]–[20]. Although our results have been obtained *in vitro* and in neonatal mice, they may be of some values for infants. Indeed, they are fully consistent with 5-HT metabolism anomalies altering breathing response to CO2/pH and increasing SIDS risks.

SERT is the primary target for widely used antidepressants. Depression concerns up to 2–3% of the pregnant women population and mothers are often subjected to treatment with SERT inhibitors, which may indirectly affect the foetuses via placental transfer and the infants via breastfeeding, resulting to clinical manifestations in 30% of the neonates, including breathing symptoms [55]. As it cannot be excluded that fluoxetine treatment of the mother depresses the infant breathing response to CO2/pH, we suggest that antidepressants targeting the 5-HT system are used with caution during pregnancy and breastfeeding period, with careful follow-up of infants exposed to these agents.
